# Developmental origins of disease – Effects of iron deficiency in the rat developing kidney and beyond

**DOI:** 10.1007/s00467-025-06762-w

**Published:** 2025-04-12

**Authors:** Anthony Babu, Whitley N. Hulse, Matthew W. Harer, Keri A. Drake, Pamela J. Kling

**Affiliations:** 1 Division of Neonatology, University of Wisconsin-Madison Department of Pediatrics, Madison, WI USA; 2 Department of Pediatrics, University of Texas Southwestern, Dallas, TX USA; 3 Unitypoint Health Meriter, 202 S. Park St., Madison, WI 53715 USA

**Keywords:** Congenital iron deficiency, Glomerulogenesis, Nephron endowment, Hypertension, Maculae densa, Developmental programming

## Abstract

Iron is an essential cofactor in metabolic and developmental processes. Iron deficiency (ID) is the most common micronutrient deficiency in pregnancy, especially impacting medically underserved populations worldwide. Iron deficiency (ID) in pregnancy predisposes neonates to poor iron status, i.e., congenital ID and associated adverse effects. The role of congenital ID on human kidney development is unstudied, but impaired fetal kidney development is possible. Both vascular and global nutrient restriction rat models report impaired fetal kidney development, as well as induce hypertension, supporting the developmental origins of health and disease (DOHaD) hypothesis. This review compiles findings from 17 published studies in rats examining congenital or early postnatal ID, showing the same. The review compares histological and physiological findings in both congenital and postnatal ID, placing these in the context of recent knowledge describing molecular mechanistic pathways regulating nephrogenesis. Findings in rat early-life ID include lower kidney iron levels, lower glomerular generations and estimated glomerular numbers, larger maculae densa size, interstitial fibrosis, and prolonging active glomerulogenesis past normal temporal cessation. Additionally, several physiological studies in rat congenital ID promote altered renin–angiotensin signaling and hypertension with maturation, especially in males. Key findings of morphological kidney maldevelopment, altered renin–angiotensin signaling, and hypertension in early-life ID underscore the urgent need for future mechanistic data in animals such as rats. The long-term goal would be to leverage understanding from these data into either preventative or early therapeutic strategies in children.

**Graphical Abstract:**

**Figure:**
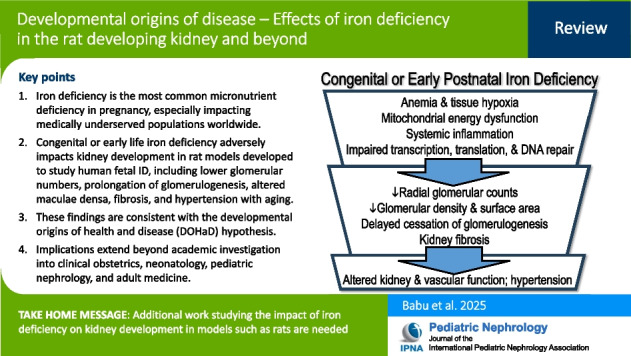
A higher resolution version of the Graphical abstract is available as Supplementary information

**Supplementary Information:**

The online version contains supplementary material available at 10.1007/s00467-025-06762-w.

## Introduction

Iron is an essential micronutrient, playing critical roles from the functional level of individual cells to regulating complex physiology in organisms. Specifically, iron is known to regulate cellular processes and homeostasis, including cellular oxygen delivery, mitochondrial energy generation, nucleotide synthesis, and DNA maintenance [1]. Iron metabolism is also functionally critical in fetal growth, organ development, and fundamental physiological roles. While iron deficiency (ID) in pregnancy is the most common micronutrient deficiency worldwide, severe ID not only results in adverse maternal effects but also results in low fetal iron stores at birth, i.e., congenital ID [1, 2]. Due to challenges in studying humans, rats have been used to model human congenital ID [1], especially studying brain maldevelopment. In addition to maternal dietary ID, other human pregnancy complications, including maternal hypertension, maternal diabetes, multifetal gestation, placental insufficiency, or fetal growth restriction, all impede placental iron transfer and may cause congenital ID [2]. Premature birth also predisposes to development of ID in the neonatal intensive care unit (NICU) when kidney development is ongoing. While the importance of iron in cellular metabolism has long been recognized, its mechanistic impact on fetal kidney development and physiology remains relatively understudied. This is despite a growing understanding of how vascular hypoxia and global fetal nutrient restriction impair kidney anatomical development and promote hypertensive vascular dysfunction in the context of the developmental origins of health and disease (DOHaD) hypothesis [3, 4]. The DOHaD hypothesis asserts that essential adaptations during deficient prenatal nutrition become maladaptive once the postnatal nutritional environment is abundant. Thus, it highlights the need to use animal models to better understand this pathophysiology to help develop specific, timely interventions to treat and/or prevent such deleterious adaptations in humans.

## Developmental origins of adult disease — DOHaD

In humans, preventing the massive global public health cost burden of treating chronic kidney disease (CKD) and hypertension necessitates strategies for prevention. Prevention strategies include a better understanding of the underlying etiologies causing disordered kidney development and strategies to optimize nephron allotment [3]. Glomerulogenesis ends around 35 weeks of gestation in humans, before normal term birth. Once glomerulogenesis is complete, no new glomeruli form with only future loss possible. Premature human infants in the NICU may experience postnatal glomerulogenesis for up to 2–3 months during which repetitive physiological and/or pharmacological insults may interfere with glomerulogenesis [5, 6]. Thus, further study is urgently needed into the critical developmental windows during which nutritional insults impact kidney development [4]. In the DOHaD field of research, both vascular hypoxia and global fetal nutritional restriction cause fetal growth restriction and promote kidney maldevelopment resulting in hypertension later in life [4]. However, limited available data have prevented a broader recognition of ID as an insult under the DOHaD umbrella. Thus, further mechanistic studies to understand the molecular pathways underlying developmental kidney dysfunction in congenital ID are necessary and remain an unmet need in addressing this potential disease burden.

## Rat models to study congenital ID

Due to the challenges of studying ID and human fetal kidney development, rat models have been leveraged for several reasons. First, physiological studies in rat congenital ID models confirm the development of adult hypertension [7–12], although blood pressure may initially measure lower before rising [7, 10]. Next, iron physiology and nutritional modeling of ID in rats is more readily accomplished than in mice, noting that high iron levels in standard laboratory rat diets may delay fetal effects until moderate maternal anemia occurs. Fetal ID can be accomplished by either feeding a moderately low iron pregestational diet through gestation and/or lactation or a markedly lower iron diet during gestation and/or lactation. Next, rats undergo postnatal nephrogenesis, which confers the advantage of an ex vivo experimental window to study the impact of ID on late nephrogenesis. Finally, rats and mice recapitulate many aspects of kidney development, but with some differences. Rats undergo two of the three phases seen in human nephrogenesis; early branching and arcade formation. Rats have the advantage over mice, which share only one phase with humans [13, 14].

Rat congenital ID models [7–10, 12, 15–19] generally initiate the ID insult during active nephrogenesis, traversing most glomerulogenesis, while a postnatal ID model [20] primarily insults during glomerular maturation and renal tubular elongation.

**Fig. 1:**
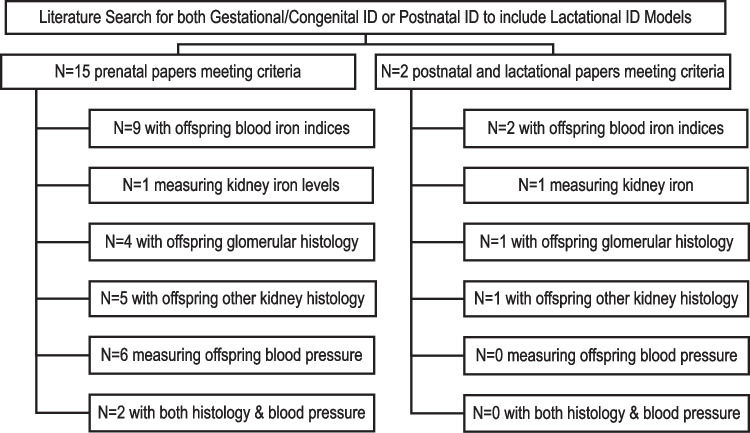
Literature search for both congenital and postnatal ID models; terms included** (**1) rat, (2) iron deficiency and ID, (3) anemia, (4) renal, (5) kidney, (6) development, (7) fetal, (8) neonatal, and (9) suckling. Furthermore, it included (1) body and renal or kidney morphometry, (2) blood hemoglobin or iron measures, (3) tissue iron measures, (4) renal or kidney histology, (5) glomerulogenesis or nephrogenesis, (6) renal or kidney fibrosis, (7) blood pressure, (8) hypertension, (9) renal or kidney failure, or (10) CKD (chronic kidney disease)

We searched the scientific peer-reviewed literature for any controlled rat ID studies modeling kidney development and/or CKD. Many rat models of early life ID were developed primarily to study either hematopoietic or brain development [1, 21–25]. Some models focused on placental or embryonic development [26, 27]. Using PubMed and Web of Science databases, we searched articles from the last 30 years that examined either congenital/gestational ID or early postnatal/lactational ID and rat kidney development. We included initial search terms that included (1) rat, (2) iron deficiency and ID, (3) anemia, (4) renal, (5) kidney, (6) development, (7) fetal, (8) neonatal, (9) suckling, and (10) early postnatal. We further searched these publications for measurements of (1) body and renal or kidney morphometry, (2) blood hemoglobin or iron measures, (3) tissue iron measures, (4) renal or kidney histology, (5) glomerulogenesis or nephrogenesis, (6) renal or kidney fibrosis, (7) blood pressure, (8) hypertension, (9) renal or kidney failure, or (10) CKD. Flow diagram, Fig. 1, summarizes the congenital/gestational and postnatal/lactational models meeting the literature search and the measurements. We searched all strains of rat models, including varied dietary iron regimens and different study endpoints. We included controlled studies of ID-induced during rat pregnancy and by feeding iron-restricted diets through pregnancy and all or part of lactation [7–12, 15–19, 25–27]. For postnatal/lactational suckling rat ID models we searched using the same parameters as gestational/congenital ID but focused on postnatal onset of ID [20, 28]. A single additional study was included for adding molecular mechanistic data pertinent to kidney developmental pathways in iron-deficient embryos [29, 30]. We found multiple manuscripts from some groups, with some reporting different data from the same model and others altering models in subsequent manuscripts. All 17 manuscripts meeting criteria were included.

**Fig. 2:**
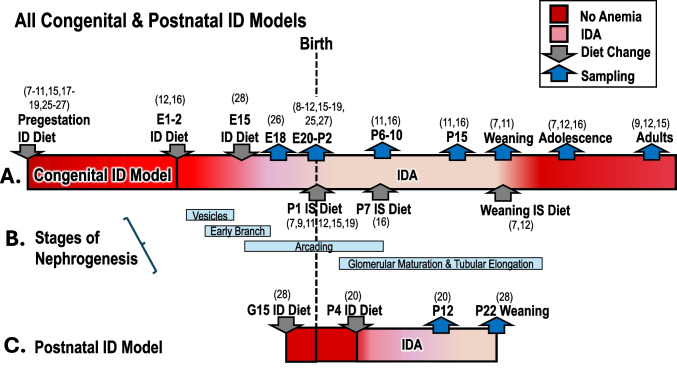
Timeline congenital and postnatal ID models; **A** congenital ID, pregestation, embryonic days (E), and postnatal days (P), top panel; **B** light blue corresponds to stages of nephrogenesis, middle panel; **C** postnatal ID; late gestational (G) and postnatal (P), lower panel. Dark red, iron sufficiency (IS); pink, iron deficiency (ID); gray down arrow, ID diet; gray up arrow, IS diet; blue up arrow, tissue and/or blood sampling; and vertical dashes, birth. Numbers in parentheses correspond to reference numbers

The intent of the current review is to determine whether these rat studies may help explain the maternal–fetal physiology behind conflicting human data. Available human studies on childhood BP based on defining either maternal ID or congenital ID were limited. For example, one review [31] compared human epidemiological studies finding that maternal hemoglobin, a meager surrogate for ID in pregnancy [32], was either directly, indirectly, or not associated with BP in pre-adolescent children. Studies meeting criteria had diverse study designs, lack of standardized histological methodology, with variable number of animals including fewer with kidney histology or physiological measures in adult offspring than with embryos or at birth. Supplementary Table 1 includes the studies’ details, including rat numbers reported. Figure 2 describes the timing of the congenital and postnatal ID models; A. top panel shows congenital ID; C. bottom panel shows postnatal ID model, both showing initiation of ID diet, gray arrows, and blue arrows the sampling, and B. middle panel shows the stages of nephrogenesis.

## Morphology and erythropoiesis in rat congenital ID models

Due to variation in study measurements, comparisons are performed by converting each continuous measure into categorical parameters of either yes/no or whether the measure is higher, lower, or the same as each study’s respective age-matched, healthy controls, as shown in Table 1. We found that most identified eligible studies utilized congenital ID models. Kidneys were studied at variable postnatal ages, from birth to adult. Models were designed such that maternal ID with anemia [7–10, 12, 15–19] and thus presumed fetal hypoxia began either prior to, in early, or in mid-gestation (Table 1A). Lower body weights and/or growth rates were reported in most rat congenital ID models [7, 9–12, 15, 18, 26, 27] (Table 1B). Some rat congenital ID models [8, 16, 26, 27] reported similar relative wet kidney weights (mg/g rat). Note that wet weights are measured without a desiccant step to remove fluid. Others [9, 10, 12, 18, 19] reported lighter relative kidney weights. Additionally, two studies found heavier relative kidney weights [7, 15] in later life (Table 1B), and, when combined with the appear

ance of edematous tubules and interstitium, these findings could suggest hyperfiltration [33].

**Table 1:** Comparison of rat ID models

Measures vs. matched-control (reference numbers in parentheses)	Congenital ID	Postnatal ID rats
	All congenital ID models [7–12, 15–19, 25–27, 29]	Postnatal ID model [20, 28]
A. Duration of ID		
Onset of ID diet	Pre-, early-, mid-gestation	Postnatal day 4 diet or onset postnatal
B. Growth/morphology		
Body weight g	↓ (7, 9–12, 15–18, 27)	↔ (20) ↓ (28)
Tail length cm	↓ (16)	↓ (20)
Kidney weight g	↓ (9, 16)	↑ (20)
Relative kidney weight mg/g rat weight	↓ **(**9**–**10**, **12**, **18**–**19**)** ** ↔ (**8**, **16**, **26**, **27**) ↑ (**7**, **15**)**	**↑ (**20**)**
C. Blood measures		
Anemia	Yes (7–12, 16–18)	Yes (20, 28)
Iron deficiency	Yes (8, 9, 12, 16)	Yes (20, 28)
C-reactive protein/inflammation	**↓ (**16**) ↑ (**25**)**	**↑ (**20**)**
D. Kidney Fe		
Kidney [Fe] mg/g wet weight	↓ (16)	↓ (20)
Kidney Fe Total mg	↓ (16)	↓ (20)
E. Glomerular histology		
Glomerular density per µm^2^	↑ (10, 16, 19)	↓ (20)
Glomerular size µm^2^	↔ (10, 16)	↑ (20)
Total glomerular surface area µm^2^	↓ (16, 19, 26)	↓ (20)
Radial glomerular counts	↓ (16)	↓ (20)
Renal cortical depth µm	** ↔ (**10**), ↓ (**16**)**	** ↔ (**20**)**
Delayed cessation glomerulogenesis	Yes (16)	Yes (20)
F. Other kidney histology		
Maculae densa cell number	**↑ (**16**)**	**↔ (**20**)**
Type 1 collagen/fibrosis	↑ (8, 10, 12, 16)	↑ (20)
G. Hypertension		
Systolic blood pressure adolescence	↓ (7, 9-females, 16)	n/a
Systolic blood pressure adults	↑ (7, 10–12)	n/a

Continuous data from rat studies converted to yes/no or arrow direction vs. control

Bolded rows show differences between gestational and postnatal models

*ID*, iron deficiency; *E*, embryonic day; *n/a*, measurement not assessed

Rats with congenital ID were generally born with the hallmark signs of severe anemia (Table 1C) [7–12, 16–18], supporting the presumption of fetal hypoxemia. Some reported liver biochemical and/or plasma measures of ID (Table 1C) [8, 9, 12, 16]. Two studies measured kidney iron content by direct iron [20] or nonheme iron [16] reported relatively lower kidney iron levels (Table 1D).

## Kidney histology in rat congenital ID

Some congenital ID studies reported histological examination using fixed specimens [10, 12, 16, 19], including most with image capture and quantitation software for structural measurements. One congenital ID study reported electron microscopy [26] (Table 1E). Microscopic histological images estimated total glomerular surface area, glomerular mass density in cortex, and/or cortical depths [10, 16, 26]. One rat congenital ID study [16] found a lower cortical glomerular density, but one study found increased density [10] (Table 1E). Three rat congenital ID studies [16, 19, 26] found lower total glomerular surface area (Table 1E). Two rat congenital ID studies [10, 16] found a smaller mean glomerular surface area (Table 1E). One congenital ID study [16] found decreased radial glomerular count (RGC) generations in a method previously reported in human [5] and primate studies [34] (Table 1E). One congenital ID study found decreased cortical depths [16], while another [10] reported unchanged cortical depth (Table 1E). Cessation of glomerulogenesis in rats occurs at postnatal (P) days 7–8 in rats. One congenital ID study [16] assessed the timing of cessation of active glomerular formation by identifying S- and C-shaped bodies in the nephrogenic zone (Table 1E), finding a delay in cessation in congenital ID rats beyond simultaneous controls at P7–8, and although the 2-day window for delivery was E21–22, residual glomerulogenesis past P10 was only observed in gestational ID rats [16, 20], a finding that should be studied with molecular markers of nephron progenitor cells.

One congenital ID study reported [16] more cells in the maculae densa (Table 1F), part of the juxtaglomerular apparatus that regulates renin secretion and controls blood pressure.

It is known that cell numbers in individual macula densa rise with angiotensin receptor blockade in adult rats [35]. We found no other rat congenital ID studies examining maculae densa cells but found studies indicating disordered renin–angiotensin system signaling in adult rats born after congenital ID [12, 15].

Four rat congenital ID studies [8, 10, 12, 16] reported either kidney fibrosis or kidney hypoxia inducible factor-1a (HIF-1a), a transcription factor regulating the profibrotic transforming growth factor-b (TGF-b) (Table 1F). Kidney fibrosis can indicate progressive kidney disease, with anemia and presumed hypoxia regulating pro-fibrotic effects of HIF-1a through tumor necrosis factor-a and interleukin-6 [36]. Placental and/or fetal kidney oxidant markers were elevated in ID [12, 18, 25, 26], although kidney HIF-1a did not differ from control in an earlier study [8] (Table 1F).

For comparisons, it would be optimal to develop a more standardized semi-quantitative scoring research methodology assessing kidney microscopic morphology to better compare animal studies examining the impact of ID on kidney development and to compare kidney microscopic morphology with both mechanistic and molecular studies. Semiquantitative methods have previously been used to assess glomerular and tubular hypertrophy and glomerular and tubular lesions in adult CKD rats [37]. In the included rat congenital or postnatal ID manuscripts, some reported (1) glomerular generations, (2) glomerular size, and (3) glomerular density [10, 16, 20], but we found no standardized approach to developmental kidney pathology. To address this limitation, future work could employ a more standardized scoring method previously reported in another species including 10 different pathological markers and nephrogenic zone width as a surrogate to measuring a delayed cessation of glomerulogenesis [38]. Alternatively immature:mature glomeruli ratios could be used to assess cessation of glomerulogenesis [39]. Exhaustive nephron counting or the dissector microscope counting [40] have also been reported but may be time or cost prohibitive.

## Physiological measures in rat congenital ID

Several congenital ID models in rats found higher systolic blood pressures (SBP) in adults [7–10, 12], some finding higher SBP in males only [9] or studying only male rats [12]. SBP was inversely related to glomerular endowment in adult rats after congenital ID [10]. Although one congenital ID study [16] reported lower SBP at P45 adolescence, the SBP may be measured too early for hypertension to develop since two other studies [7, 9] also found lower SBP early in postnatal life, followed by higher SBP later. It is also possible that hypertension responsiveness to ID differs between rat strains because gatekeeper genes that drive developmental programming differ between strains [26].

## Defining critical windows and therapeutic implications by comparing rat congenital and postnatal ID

Gaps exist in our understanding of hypertension in the context of the DOHaD hypothesis that animal models of rat ID can address. To prevent hypertension and its sequelae, future work should define the critical vulnerable developmental window that directly leads to microscopic lesions and/or altered gene expression impacting the development of hypertension in either rat congenital ID or rat early postnatal ID. Our understanding of the developmental window can be affected by varying either the timing of the introduction or severity of the ID diet. Next, it is necessary to define what developmental window is also responsive to specific hypertension preventative or therapeutic strategies.

Only one center performed both congenital and postnatal ID models, allowing direct comparison of these models. In congenital ID, the diet started at E2, but reached maternal ID with anemia at mid-gestation early branching and arcading [16] and a rat postnatal ID model started at P4 covering largely glomerular maturation and tubular elongation [20]. Due to the immaturity of term rats, this postnatal ID model was designed to study human prematurity [20]. From this center, relative wet kidney weights (mg/g rat) in congenital ID did not differ from control [16], while the postnatal ID had heavier relative wet kidney weights (mg/g rat) than control [20]. Note that wet weights are measured without a desiccant step to remove fluid (Table 1). Heavier relative wet kidney weights, although not confirmed, potentially implicate either hypertrophy or early hyperfiltration [33]. Because anemia and decreased tissue oxygen delivery typically slow growth rates, overworked nephrons in the rat postnatal ID model may promote tissue hypoxia, hyperfiltration and/or inflammation [33]. This observation is noteworthy because a goal in caring for premature babies is to match typical intrauterine growth rates. Comparing the congenital and postnatal models from the same center [16, 20], lower kidney iron content was seen in both (Table 1), linking tissue ID as a direct contributor to altered microscopic development. Congenital ID [16], with smaller mean glomerular size and cortical depth, differed from postnatal ID, with larger mean glomerular size and unchanged cortical depth [20] (Table 1). Larger glomerular size may potentially implicate hypertrophy or hyperfiltration [33]. Both congenital and postnatal models [16, 20] exhibited lower radial glomerular counts and prolongation of glomerulogenesis beyond P7–8 (Table 1), a finding noteworthy because only loss is possible once glomeruli are formed. Whether prolonging glomerulogenesis is an adaptive process to increase functional numbers or a maladaptive process with the development of dysfunctional glomeruli is unknown but should certainly be studied. We found no rat studies describing prolonged nephrogenesis. Although there was evidence for postnatal continuation of glomerulogenesisis in premature humans, prolonging of nephrogenesis was not observed in autopsy specimens of kidneys from preterm infants with prolonged NICU stay compared to controls after early post-birth death [6] or in autopsy specimens from all gestational ages deceased in utero or immediately post-birth [41]. Delayed molecular control of the cessation of normal nephrogenesis beyond its developmental window is of much interest in premature infants [42] for its potential to improve nephron endowment through therapeutic protein, fat, vitamin, or other pharmacotherapy intervention within the NICU [18, 19].

Only two studies investigated maculae densa numbers, finding that higher cell numbers in individual maculae densa were seen in congenital ID [16] but not postnatal ID [20] (Table 1). Maculae densa are juxtaglomerular cells that control renin secretion, alter renin feedback mechanisms, and contribute to hypertension [12, 15, 43]. Higher cell numbers of individual maculae densa cells were also observed in adult rats treated with angiotensin receptor-1 blockade [35]. Comparing interstitial fibrosis, it was seen in both rat congenital ID [16] and postnatal ID models [20] (Table 1). Fibrosis often develops as an inflammatory response through HIF-1a-regulated TGF-b activation of fibroblasts, extracellular collagen deposition, and/or as a response to hyperfiltration. Linking inflammation and/or hyperfiltration to SBP was not performed in our rat postnatal ID model [20] but should be performed. The only other identified study of postnatal ID model used a lactational ID model [28] designed to lower milk iron levels for ID to present postnatally, but did not study kidneys. This study [28] was included for comparison despite the absence of offspring kidney measures published or unpublished (through communication by Dr. EL Unger on 5/15/2024) because the model may have utility for kidney studies as it reported smaller rats, anemia, ID, lower liver and spleen iron, and systemic inflammation [28], similar to congenital ID models.

## Gaps and advances in the molecular understanding of ID and glomerulogenesis

Molecular pathways behind ID and glomerulogenesis were unknown when most studies linking rat congenital ID to abnormal kidney development or hypertension were published [7, 9–11, 15, 16, 20]. However, recent physiologic studies promote understanding of organogenesis [12, 26, 30, 42, 44–47] and provide a recent advancement in the molecular understanding of glomerulogenesis [42, 48–57]. Current tools include genetic mouse models, cell signaling, epigenetics, transcriptomics, and gene ontology analyses that help to address gaps in our understanding of the impact of ID on glomerulogenesis.

Although absolute glomerular numbers are highly variable between individuals, low glomerular numbers are linked to hypertension in human and animal models [9, 11, 12, 19, 56]. Examining glomerular numbers, windows during active glomerulogenesis, number of individual maculae densa, and/or interstitial fibrosis in the context of newer mechanistic literature would be informative. Recent work shows the imbalance between GDNF and WNT systems may be responsible for controlling the cessation of glomerulogenesis [53, 58], which should be studied in the context of ID.

ID may also interfere with hormonal metabolism necessary for glomerulogenesis because iron is an essential cofactor that either transports the active hormone or activates hormones needed for normal nephrogenesis, including (1) vitamin A/retinoic acid, (2) vitamin D/calcitriol, and (3) growth hormone/insulin-like growth factor-1 and its binding proteins [19, 38, 59]. First, recent work implicates congenital ID in disrupting crucial retinoic acid signaling during nephrogenesis [17–19, 27] and should be further studied. Next, congenital vitamin D-deficient rat models showed smaller but a greater number of glomeruli and a higher immature:mature glomerular ratio [39, 59], an adaptation that may partially normalize the glomerular filtration rate. These studies [39, 59] support the investigation of kidney vitamin D status in the context of congenital ID because depleted iron can alter heme-containing enzymes necessary for activating vitamin D. Additionally, insulin-like growth factor (IGF)−1 signals growth pathways, and IGF-1 supplementation may improve nephrogenesis [38]. Iron is known to regulate IGF-1 and several other growth factors, kinases, and transcription factors involved in growth and cell cycle regulation during nephrogenesis, including ERK, PI3K, RET, mTOR, and Six2 [26, 29, 60].

Differentially regulated genes were found in placental and whole embryos after rat congenital ID [26, 29, 60], but glomerulogenesis was not studied. One study of congenital ID found both altered GDNF kidney developmental pathways and deficient vitamin A–retinoic acid pathways in kidney tissue that also accompanied reductions in nephron endowment [19].

Using genetic mouse models, WNT 11 and GDNF regulate nephron progenitor cell lifespan and nephron endowment [42, 61] and could guide understanding of how iron may control molecular pathways regulating nephrogenesis and nephron progenitor cells. Knock-out models for critical iron transporters are embryonically lethal, and conditional models are needed to study iron and nephrogenesis. A heterozygous transferrin receptor-1 mouse model showed normal early kidney development but in adulthood found attenuated TGF-b receptor-1 and Smad2 signaling and greater fibrosis post fibrotic stimulus [62]. Thus, within the framework of DOHaD, the development of hypertension may need a second insult, such as a fibrotic stimulus.

Although the published papers described herein report an association between ID and hypertension, the papers are limited in that there is no defined direct link to causality. Observations involving the renin–angiotensin pathway in rat congenital ID include a greater number of individual maculae densa cells [16] and altered signaling in renin–angiotensin pathways [12] that are linked to hypertension risk. Notably, cell number in individual maculae densa also rose in angiotensin type 1 receptor blockade [35], supporting iron’s potential role in the renin–angiotensin pathway. In addition, observations of increased kidney fibrosis [16, 20] and altered signaling pathways involving cytokines, oxidative stress, and mitochondrial respiration in fibrosis [12, 17, 18] in early life ID are noteworthy. ID upregulates kidney HIF-1a [8, 25], a transcriptional regulator of TGF-b that regulates epithelial-to-mesenchyme transition and tubulointerstitial fibrosis [63]. Understanding the genetic control of kidney fibrosis in ID is important because of findings that altered Notch, WNT, and Sonic hedgehog pathways in genetic models may promote kidney fibrosis [61].

## Conclusions, lifelong consequences, and future directions

Combining well-established physiological rat ID models with additional molecular mechanistic studies may advance the understanding of altered developmental kidney histology and physiology. Despite differences in the timing and duration of dietary congenital ID models and using several strains of rats, microscopic and physiological findings were relatively consistent. Adult rat offspring of both sexes after congenital ID had higher SBPs, although males may be more vulnerable. However, because some models included only males or had limited power to examine sex differences, this should be studied more thoroughly. With advances in molecular mechanistic understanding of kidney development, physiological rat ID models are poised to address the impact of ID on the regulation of glomerular endowment, cessation of nephrogenesis, altered number of maculae densa cells and renin–angiotensin signaling, and kidney fibrosis. Altering the timing of insult in models should address critical developmental windows, including either preventive interventions or potential therapeutic windows that may prevent hypertension and improve long-term kidney outcomes, including blood pressure. Although this manuscript is of interest to neonatologists, pediatric nephrologists, and adult nephrologists, the research papers summarized in this report are based on animal models and lack direct clinical application. Although these findings provide key insights into understanding the impact of ID during pregnancy on fetal kidney development, translating these findings into clinical practice remains a challenge.

## Key points

1. *Iron deficiency (ID)* adversely impacts kidney development in rat models of congenital or postnatal ID, consistent with the developmental origins of health and disease (DOHaD) hypothesis.
2. *Both congenital ID and postnatal ID* altered kidney development, with relatively lower radial glomerular counts and estimated glomerular numbers, and more fibrosis.
3. *Kidney development in a postnatal ID model in rats*, essentially a model of prematurity, was designed to maintain mean growth rate and led to relatively larger glomeruli, but normal maculae densa.
4. *Both rat congenital ID and postnatal ID models showed delayed cessation of active glomerulogenesis*, including immature microscopic histology implicating an adaptation to prolong glomerulogenesis.

## Supplementary Information

Below is the link to the electronic supplementary material.

Graphical abstract (PPTX 77 KB)

Supplementary file2 (XLSX 12 KB)

## Data Availability

Data sharing is not applicable to this article as data are published with no new datasets generated or analyzed in the current paper.
