# Supplementary material for: Developmental origins of disease – Effects of iron deficiency in the rat developing kidney and beyond
Source: Pediatr Nephrol. 2025 Apr 12;41(1):23–32. doi: 10.1007/s00467-025-06762-w (PMC12686096; doi:10.1007/s00467-025-06762-w)
Supplement: Supplementary file 1 — Graphical abstract (PPTX 77 KB) [file 467_2025_6762_MOESM1_ESM.pptx]

## Slide 1
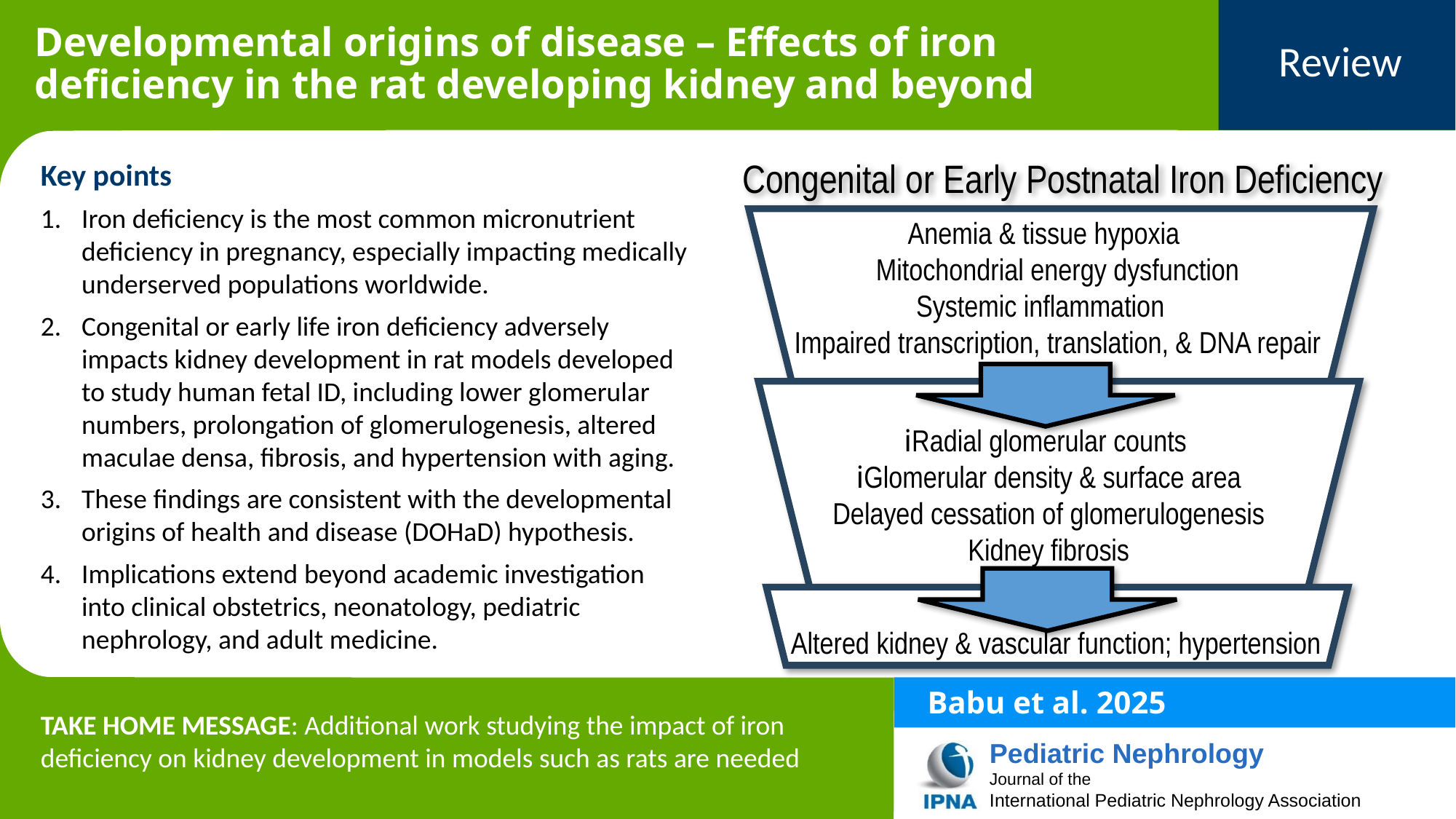

Developmental origins of disease – Effects of iron deficiency in the rat developing kidney and beyond
Congenital or Early Postnatal Iron Deficiency
Key points
Iron deficiency is the most common micronutrient deficiency in pregnancy, especially impacting medically underserved populations worldwide.
Congenital or early life iron deficiency adversely impacts kidney development in rat models developed to study human fetal ID, including lower glomerular numbers, prolongation of glomerulogenesis, altered maculae densa, fibrosis, and hypertension with aging.
These findings are consistent with the developmental origins of health and disease (DOHaD) hypothesis.
Implications extend beyond academic investigation into clinical obstetrics, neonatology, pediatric nephrology, and adult medicine.
Anemia & tissue hypoxia
Mitochondrial energy dysfunction
Systemic inflammation
Impaired transcription, translation, & DNA repair
iRadial glomerular counts
iGlomerular density & surface area
Delayed cessation of glomerulogenesis
Kidney fibrosis
Altered kidney & vascular function; hypertension
Babu et al. 2025
TAKE HOME MESSAGE: Additional work studying the impact of iron deficiency on kidney development in models such as rats are needed
